# Results of noninvasive ventilation in very old patients

**DOI:** 10.1186/2110-5820-2-5

**Published:** 2012-02-21

**Authors:** Frederique Schortgen, Arnaud Follin, Lucilla Piccari, Ferran Roche-Campo, Guillaume Carteaux, Elodie Taillandier-Heriche, Sebastien Krypciak, Arnaud W Thille, Elena Paillaud, Laurent Brochard

**Affiliations:** 1 AP-HP, Groupe Hospitalier Albert Chenevier-Henri Mondor, Réanimation Médicale, Créteil, France; 2 INSERM, U955, Faculté de Médecine, Créteil, France; 3 AP-HP, Groupe Hospitalier Albert Chenevier-Henri Mondor, Unité de médecine gériatrique, Créteil, France; 4 Université Paris Est, Faculté de Médecine, Créteil, France; 5 Soins Intensifs, Hôpital Universitaire, University of Geneva, Geneva, Switzerland

## Abstract

**Background:**

Noninvasive ventilation (NIV) is frequently used for the management of acute respiratory failure (ARF) in very old patients (≥ 80 years), often in the context of a do-not-intubate order (DNI). We aimed to determine its efficacy and long-term outcome.

**Methods:**

Prospective cohort of all patients admitted to the medical ICU of a tertiary hospital during a 2-year period and managed using NIV. Characteristics of patients, context of NIV, and treatment intensity were compared for very old and younger patients. Six-month survival and functional status were assessed in very old patients.

**Results:**

During the study period, 1,019 patients needed ventilatory support and 376 (37%) received NIV. Among them, 163 (16%) very old patients received ventilatory support with 60% of them managed using NIV compared with 32% of younger patients (*p *< 0.0001). Very old patients received NIV more frequently with DNI than in younger patients (40% vs. 8%). Such cases were associated with high mortality for both very old and younger patients. Hospital mortality was higher in very old than in younger patients but did not differ when NIV was used for cardiogenic pulmonary edema or acute-on-chronic respiratory failure (20% vs. 15%) and in postextubation (15% vs. 17%) out of a context of DNI. Six-month mortality was 51% in very old patients, 67% for DNI patients, and 77% in case of NIV failure and endotracheal intubation. Of the 30 hospital survivors, 22 lived at home and 13 remained independent for activities of daily living.

**Conclusions:**

Very old patients managed using NIV have an overall satisfactory 6-month survival and functional status, except for endotracheal intubation after NIV failure.

## Introduction

The use of noninvasive ventilation (NIV) as first-line supportive therapy for acute respiratory failure (ARF) is increasing in the ICU. The reduced invasiveness of this technique in selected populations of critically ill patients leads to better outcomes than with endotracheal intubation. NIV reduces the need for intubation and decreases mortality during acute-on-chronic respiratory failure (AOC), cardiogenic pulmonary edema (CPE), and *de novo *ARF in immunocompromised patients [1-6]. Recently, NIV has been proposed for the prevention of postextubation ARF for at-risk patients, with promising results [7,8]. The choice of NIV aims to avoid complications, particularly in fragile patients [9]. Patients aged 80 years or older, also referred to as "very old patients," are potentially "good candidates" for a less invasive management.

The proportion of elderly persons among hospitalized patients, including ICU admissions, is rapidly growing in developed countries. In recent epidemiological studies, very old patients represent 10-15% of ICU admissions [10-12]. Also, the incidence of ARF increases exponentially with age [13]. Elderly patients are particularly susceptible to chronic heart failure and pulmonary diseases, which are classical causes of respiratory failure needing ICU admission [14]. The management of critical respiratory illness in the elderly is therefore of particular importance. NIV also is frequently proposed for the respiratory support of patients with a do-not-intubate order (DNI), as supported by the results of a recent, randomized controlled study and surveys [15-18]. Although DNI in itself cannot be considered as an indication of NIV, the place of NIV as a ceiling therapy or a comfort treatment for patients with acute respiratory failure near the end of life is debated and a better delineation of the place for palliative NIV among overall indications of NIV is needed [19]. In this context, we sought important to isolate NIV performed in the context of DNI.

Whereas NIV is an attractive technique for the management of ARF in very old persons, specific data for this population are limited, especially for long-term mortality [20-23]. The goal of this prospective cohort study was to identify the conditions in which NIV is applied to very old patients in the ICU compared with younger patients and to assess its influence on long-term outcome and functional status.

## Methods

### Setting and population

The study was approved by the Ethics Committee of the French Society of Intensive Care Medicine (n° 08-260). According to the French legislation, requirement to obtain written informed consent was waived. During ICU stay, patients or their surrogates were informed about data collection for the research and about their right to refuse. Information about their right to refuse also was specified at time of phone interview.

The study was conducted in the 24-bed medical ICU of the Henri Mondor University Hospital. Although NIV can be started outside of the ICU, patients needing NIV for ARF are usually admitted to our ICU. In this closed unit, NIV is managed by ICU physicians and nurses in charge of the patient. ICU ventilators with oronasal or total face masks are used for NIV sessions. Pressure support mode is applied as the first choice. Arterial blood gases are usually measured after 1 hour of treatment to assess the response to NIV and to modify the settings accordingly. Due to the growing use of NIV in our unit, a specific registry for patients who undergo at least 2 hours of NIV had been implemented [2,24].

The investigator in charge of registry completion was not involved directly in patient care. In the registry, severity at admission and at the start of NIV is assessed by SAPS II [25] and SOFA [26] scores. Patients are prospectively classified according to the context in which NIV treatment is administered; cardiogenic pulmonary edema and/or acute-on-chronic respiratory failure, including COPD exacerbation (CPE-AOC), *de novo *ARF, postextubation NIV, and do-not-intubate or reintubate order (DNI) is considered as a separate group. In the case of multiple indications of NIV during the ICU stay, patients with DNI decisions were classified in the DNI group regardless of the initial cause of ARF, and non-DNI patients were classified according to the first indication. NIV failure was defined as the need for endotracheal intubation and/or a continuing need for NIV on day 6 and/or ICU death [27]. We added the continuing need for NIV to the definition of NIV failure because, in DNI patients, endotracheal intubation is not applicable. Vital status was recorded at ICU and hospital discharge.

Additional information on comorbidities and long-term outcomes was specifically recorded for very old patients included in the ICU registry from January 1, 2007 to December 31, 2008. Comorbidities were assessed using the Charlson index [28]. End-stage chronic respiratory failure definition was based on the National Hospice Organization guidelines and included at least two criteria among: O_2 _or NIV home treatment, previous ICU admission for ARF within the past year, FEV1 < 30% of predicted value, or *cor pulmonale *[29]. For each hospital survivor, vital status and living conditions were assessed by either one ICU nurse or one ICU physician (AF) through telephone interviews. The first phone contact was made with the patient's general practitioner. If this was not possible, the patient's relatives or the patients themselves were contacted. For each patient, phone contact was performed at least 6 months after ICU admission. A 10-minute interview was developed using a specific chart that included standardized questions. The patient's vital status or date of death, living location, and need for home respiratory treatments (i.e., oxygen and NIV) were recorded. Independence in activities of daily living (ADL) [30] was assessed in survivors at the time of phone interview and was used to retrospectively determine their pre-ICU status. The validated ADL system assesses the ability of patients for bathing, dressing, toileting, transfer, continence, and feeding. For each function, patient dependence was described as no help, partial assistance, and complete assistance.

### Statistical analysis

Categorical variables were expressed as percentages and continuous variables as the median and interquartile range (25^th^-75^th ^IQR). Categorical variables were compared between very old patients and younger patients, younger than aged 80 years, using the chi-square or Fisher's exact test, and continuous variables using the nonparametric Mann-Whitney test as appropriate. *P *value ≤ 0.05 in a two-tailed test was considered statistically significant. Statistical tests were performed using Intercooled STATA 8.2 software (StatCorp, Texas, USA).

## Results

### Characteristics of very old patients

**Figure 1:**
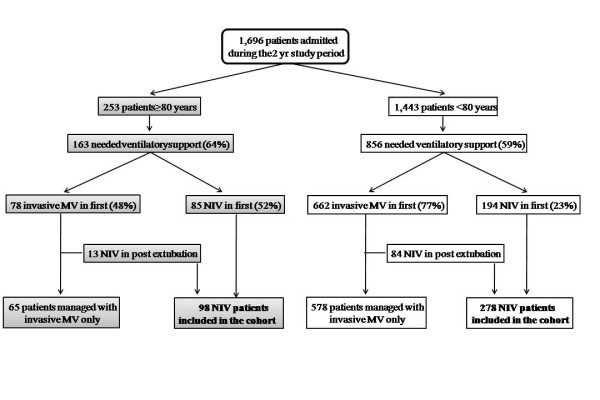
**Flow chart of the cohort study**.

During the 2-year study period, 1,696 patients were admitted to the ICU: 1,019 of these patients (60%) required ventilatory support, and 376 (37%) received NIV during their ICU stay (see flow chart in Figure 1). The proportion of patients needing ventilatory support was similar in very old (163/253, 64%) and in younger patients (856/1,443, 59%; *p *= 0.12). NIV as first-line therapy was more frequent in very old patients (85/163, 52%) compared with younger patients (194/856, 23%; *p *< 0.0001); 13 additional very old patients received NIV after extubation compared with 84 younger patients. The characteristics of the 98 very old and the 278 younger patients receiving NIV as first-line ventilatory support or after extubation during the same period are indicated in Table 1. The majority of very old patients (88%) were living at home before hospital admission; 14% had home respiratory support before admission and 18% had been previously admitted to the ICU for ARF. The most frequent circumstance for NIV use in very old patients (40%) was a DNI decision compared with only 8% in younger patients. In DNI patients, the cause of ARF was CPE or COPD exacerbation in 30/39 (77%) of the very old patients compared with 10/22 (45%) of the younger patients (*p *= 0.013). Very old patients had a significantly higher PaCO_2 _at the start of NIV and received NIV for a longer duration than younger patients.

**Table 1:** Characteristics of all patients managed with NIV according to age

	Patients ≥ 80 y (n = 98)	Patients < 80 y (n = 278)	*p *value
**Characteristics at ICU admission**			
Age, yr	84 (80-86)	67 (54-74)	< 0.001
[min-max]	[80-94]	[17-79]	
Gender, M/F, n	45/53	185/93	< 0.001
Home respiratory support, n (%)	14 (14)	28 (10)	0.4
Nasal O_2_	10	17	
NIV	4	11	
History of ICU admission for ARF, n (%)	18 (18)	49 (18)	0.87
Immunocompromised, n (%)^a^	9 (9)	54 (19)	0.02
Location before ICU admission, n (%)			0.15
Emergency room	59 (60)	140 (50)	
Medical ward	28 (29)	110 (40)	
Surgical ward	11 (11)	28 (10)	
NIV start before ICU admission, n (%)	15 (15)	28 (10)	0.16
SAPS II at admission, points	43 (36-52)	39 (31-49)	< 0.01
Non-age-related SAPS II, points^b^	25 (18-34)	27 (20-38)	0.21

**Characteristics at NIV start**			
Patients with extra respiratory organ failure, n (%)^c^	65 (66)	189 (68)	0.76
NIV context, n (%)			< 0.001
CPE-AOC respiratory failure	30 (31)	93 (34)	
*de novo *ARF	16 (16)	79 (28)	
Postextubation	13 (13)	84 (30)	
Do-not-intubate order	39 (40)	22 (8)	
ABG before NIV start			
pH	7.35 (7.27-7.42)	7.38 (7.30-7.44)	0.05
PaCO_2_, mmHg	57 (40-71)	47 (37-60)	< 0.01
PaO_2_/FiO_2_, mmHg	189 (145-235)	190 (120-240)	0.69

**NIV management**			
NIV duration within the first 24 hours, h	6 (4-10)	4 (3-8)	< 0.001
Period of NIV delivery during ICU stay, d	3 (1-5)	2 (1-3)	< 0.001
Discharged from ICU with NIV, n (%)^d^	9/94 (10)	11/267 (4)	0.05

ABG, arterial blood gases; ARF, acute respiratory failure; CPE-AOC, cardiogenic pulmonary edema and acute-on-chronic respiratory failure; ICU, intensive care unit; NIV, noninvasive ventilation. ^a^AIDS, neutropenia < 500/mm^3^, immune-suppressive drugs, chemotherapy, long-term, or recent high dose of steroids. ^**b**^Simplified Acute Physiology Score (SAPS II) minus points for age. ^**c**^At least one point in the nonrespiratory SOFA score. ^d^In patients without NIV home treatment before ICU admission.

### Survival and functional status of very old patients

**Figure 2:**
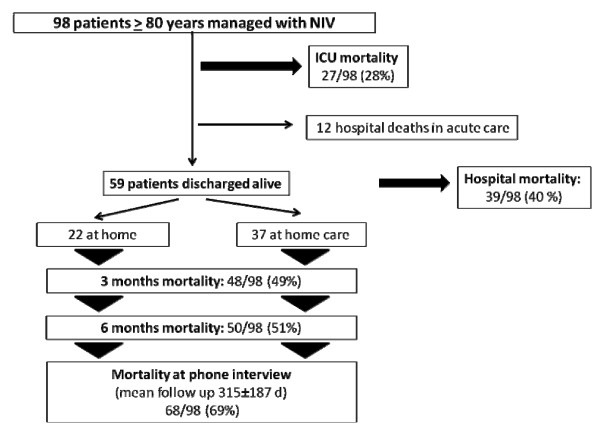
**Outcome in very old patients managed with NIV**.

The median follow-up was 316 (range, 204-391) days after ICU admission. ICU and hospital mortality were 28% and 40% respectively (Figure 2). Of the 59 very old patients discharged alive from hospital, 22 (37%) were discharged at home and 37 (63%) into a nursing home. Twenty-nine of the 59 hospital survivors died after hospital discharge with a median time of 231 (range, 136-474) days. Vital status assessment was not possible in four patients after hospital discharge; for these patients, their general practitioners thought that they were possibly dead but had no definitive information, and they were recorded as dead at 3 months. The overall 3-month and 6-month mortality rates were 49% and 51%, respectively (Figure 2).

Thirty very old patients (31%) were alive when contacted for phone interview (Table 2). Twenty-two (73%) were living at home compared with 27 (90%) before ICU admission (*p *= 0.18). An ADL score could be recorded for all 30 survivors at phone interview (Table 2). Thirteen (43%) returned to total independence in daily activities compared with 18 (60%) before ICU admission (*p *= 0.2). Only five patients were completely unable to care for themselves. The detail for each activity is indicated in Table 3. At phone interview, 12 survivors (40%) were under home oxygen (n = 8) or NIV (n = 4) compared with only two patients needing oxygen before ICU admission (Table 2). Of note, 8/10 patients on home O_2 _therapy and all 4 patients on home NIV before ICU admission were dead when contacted for phone interview [12/14 (86%) mortality for those on home respiratory support].

**Table 2:** Living conditions of the 30 survivors at phone interview (> 6 months)

	Before ICU (n = 30)	After ICU (n = 30)	*p *value
**Living**			0.2
At home, n (%)	27 (90)	22 (73)	
Home care, n (%)	3 (10)	8 (17)	

**Global functional status, n (%)**			0.2
Full function (ADL 6)	18 (60)	13 (43)	
Moderate impairment (ADL 4-5)	8 (27)	9 (30)	
Severe impairment (ADL < 2)	2 (7)	5 (17)	

**Chronic respiratory support, n (%)**			< 0.01
No	28 (93)	18 (60)	
NIV dependency	0	8 (27)	
O_2 _dependency	2 (7)	4 (13)	

ADL, activities of daily living; ICU, intensive care unit; NIV, noninvasive ventilation.

**Table 3:** Comparison of functional autonomy before and after ICU admission in the 30 survivors at phone interview according to the activities of the Katz's Activities of Daily Living (ADL) [30]

	Before ICU	After ICU	*p *value
**Bathing, n (%)**			0.052
Independent	21 (70)	14 (47)	
Partly dependent	8 (27)	9 (30)	
Dependent	1 (3)	7 (23)	

**Dressing, n (%)**			0.064
Independent	23 (77)	17 (57)	
Partly dependent	6 (20)	6 (20)	
Dependent	1 (3)	7 (23)	

**Toileting, n (%)**			0.516
Independent	26 (87)	22 (73)	
Partly dependent	2 (7)	3 (10)	
Dependent	2 (7)	5 (17)	

**Transfer, n (%)**			0.148
Independent	25 (83)	18 (60)	
Partly dependent	3 (10)	7 (23)	
Dependent	2 (7)	5 (17)	

**Continence, n (%)**			0.657
Independent	22 (73)	20 (67)	
Partly dependent	2 (7)	1 (3)	
Dependent	6 (20)	9 (30)	

**Feeding, n (%)**			0.299
Independent	27 (90)	22 (73)	
Partly dependent	2 (7)	4 (13)	
Dependent	1 (3)	4 (13)	

ICU, intensive care unit.

### Comparison of hospital outcome between very old and younger patients

**Figure 3:**
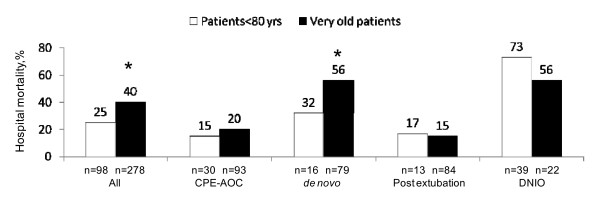
**Hospital mortality according to age and the context of NIV**. DNIO, Do-not-intubate order; CPE-AOC, cardiogenic pulmonary edema and acute-on-chronic respiratory failure. *p < 0.05 vs. younger patients.

**Figure 4:**
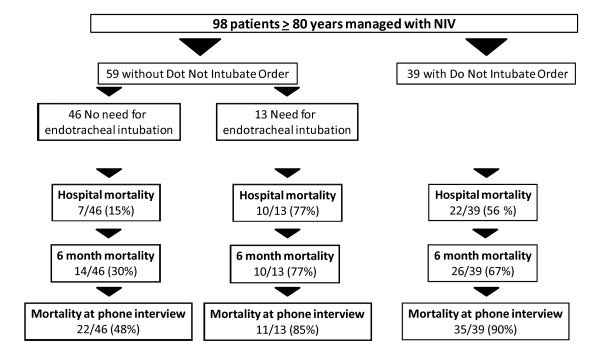
**Outcome in very old patients according to the context and success of NIV**.

**Figure 5:**
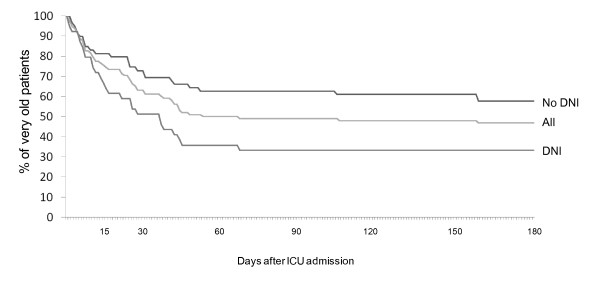
**Kaplan-Meier survival curves of very old patients after ICU admission**. DNI, do-not-intubate.

Compared with the 278 younger patients who received NIV, very old patients had significantly higher ICU and hospital mortality (28% vs. 17%, *p *= 0.03, and 40% vs. 25%, *p *< 0.01). Hospital mortality was similar in the two groups when NIV was applied for CPE-AOC respiratory failure and during the postextubation period, both out of the context of a DNI order (Figure 3). Hospital mortality was particularly high in the DNI group, both in very old (56%) and in younger patients (72%; Figure 3). Among the 39 very old patients with DNI, 10% only were alive when contacted for phone interview (Figure 4). Very old DNI patients were older, had significantly more comorbidities, and had more severe hypercapnia than very old patients with full life support (Table 4). The Kaplan-Meier survival curves according to DNI status are shown in Figure 5.

**Table 4:** Characteristics of very old patients according to do-not-intubate (DNI) status

	Very old patients without DNI order (n = 59)	Very old patients With DNI order (n = 39)	*p *value
**Decision of limitation**			NA
Do not intubate	0	33	
Do not reintubate after extubation failure	0	6	

**Age (yr)**	84 (81-85)	86 (83-89)	<0.01

**Sex Male, n (%)**	27 (46)	18 (46)	0.97

**Patients living at home before hospital admission, n (%)**	54 (92)	32 (82)	0.16

**Charlson comorbidity index, n (%)**			0.01
No or low comorbidities (0-1 point)	28 (47)	8 (21)	
High comorbidities (> 1 points)	31 (53)	31 (79)	

**Type of comorbidities**			
Dementia	1 (2)	6 (15)	0.02
Full dependency for ADL	0	11 (28)	<0.001
End-stage respiratory failure	4 (7)	16 (41)	<0.001
with home respiratory support	4	10	0.016
Active cancer	7 (12)	4 (10)	0.99
Chronic heart failure	24 (41)	17 (44)	0.77
Peripheral obstructive arterial disease	5 (8)	6 (15)	0.34

**SAPS II, points**	43 (36-52)	43 (36-50)	0.75
**Patients with extra-respiratory OF^a^, n (%)**	38 (64)	27 (69)	0.67

**ABG before NIV start**			
pH	7.36 (7.27-7.43)	7.32 (7.22-7.4)	0.18
PaCO_2 _(mmHg)	46 (38-64)	67 (53-80)	<0.01
PaO_2_/FiO_2 _ratio (mmHg)	180 (120-268)	195 (168-216)	0.59

**NIV delivery**			
Within first 24 hours (h)	6 (4-8)	9 (6-15)	<0.001
During ICU stay (d)	2 (1-3)	4 (3-6)	<0.001
Continuing need for NIV on day 6, n (%)	3 (5)	12 (33)	0.011

**ICU length of stay (d)**	8 (5-13)	8 (5-14)	0.87
Among survivors	8 (5-13)	7 (5-14)	0.48

**Hospital length of stay (d)**	25 (13-40)	20 (8-37)	0.21
Among survivors	27 (17-40)	29 (17-45)	0.25

ADL, activities of daily living; ABG, arterial blood gases. ^a^Organ failure (OF) is defined by at least one point in the nonrespiratory SOFA score.

The incidence of NIV failure was similar between very old and younger patients (42% vs. 40%, respectively). The in-hospital mortality of very old patients intubated because of NIV failure was significantly higher than for younger patients (10/13, 77% vs. 38/82, 46%, *p *= 0.01; Figure 4). Among the 13 very old patients requiring intubation, 8 received NIV for *de novo *ARF. Intubated very old patients were significantly more hypoxemic than nonintubated very old patients (P/F ratio of 110 (100-150) vs. 200 (150-300) mmHg, *p *< 0.001).

## Discussion

This cohort is the largest to date concerning NIV applied to very old patients in the ICU for ARF and shows several specific features in comparison to younger patients. Sixty percent of very old patients needing respiratory support were managed using NIV compared with only 32% of younger patients, and very old patients represented 26% of all patients managed with NIV in our ICU. NIV was applied in 40% of the very old patients with a DNI order. This large number of very old patients who received NIV observed in our ICU warranted the development of a specific long-term follow-up study. The 6-month survival rate of very old patients was 51% with satisfactory living conditions. The number of survivors needing chronic respiratory support was, however, more frequent after than before ICU admission. Hospital survival of very old patients was similar to younger patients when NIV was applied for the recommended indications, i.e., CPE-AOC respiratory failure and the prevention of postextubation ARF out of a DNI context. NIV in a context of DNI was associated with a poor outcome in both very old and younger patients.

The admission of very old patients to the ICU raises the question of the benefits and risks of invasive supportive care. In adults requiring mechanical ventilation, the likelihood of death significantly increased with age [31]. In patients aged 70 years or older, complications during the course of mechanical ventilation increased the risk of hospital mortality [32]. This suggests that avoiding invasive procedures might be particularly crucial in the elderly, even if the impact of the intensity of care on the survival of elderly patients is still under debate [33]. The greater use of NIV in very old patients than in younger found in our study could be due to physicians choosing a less invasive technique. Also, neurologic disease is less frequently the primary reason for mechanical ventilation in elderly patients and the need for ventilatory support results more frequently from respiratory distress, which represents the most frequent reason for ICU referral in very old patients [32,34].

Previous clinical trials on NIV have included some very old patients, but the median age was usually approximately 75 when studying NIV for hypercapnic ARF, and much younger in case of hypoxemic nonhypercapnic ARF [1,2,5,35]. One previous study focused on 106 very old patients who needed mechanical ventilation. The ICU mortality of NIV patients was of 21%, quite similar to that in our cohort (28%), and with a 2-year mortality of 88% [21]. Recently, Nava and coworkers reported the result of a RCT on the efficacy of NIV in patients older than 75 admitted for hypercapnic ARF [16]. In this study, 22 of 41 patients with DNI orders included in the standard medical therapy group received NIV as a rescue therapy. The mortality rate in this subgroup was comparable with the overall NIV group. The 6-month mortality of patients who received NIV was lower than in our study (27%). Patients enrolled in this RCT were limited to hypercapnic ARF and were slightly younger and had fewer comorbidities.

Hospital mortality of general populations of ICU very old patients varies from 24% to 50%, suggesting differences in the triage for ICU admission [10,12,36]. In 228 very old patients admitted in one ICU in Paris, 3-month mortality rate was approximately 50% [37]. In a cohort of 233 patients with similar age and disease severity, Boumendil et al. showed a 2-month mortality of 41% [38]. Whereas all patients needed respiratory support in our cohort, we found a comparable 3-month mortality rate (49%). We observed that deaths occurred predominantly within the first 3 months after hospital discharge, which is consistent with previous reports [37].

The majority of survivors at phone interview were living at home with little or no limitation of daily activities (Table 2), which is in accordance to previous studies showing little change in functional status after ICU discharge [36,38,39]. The evolution of functional autonomy varied according to the activity with a trend toward more dependent patients after ICU stay for bathing, dressing, and transfer. Pre-ICU status was, however, retrospectively evaluated at phone interview with a potential bias in case of functional status underestimation. Whatever the pre-ICU status, only 5 of the 30 survivors had severe functional impairment. The higher number of very old patients discharged from the ICU with the need for NIV and the higher number of survivors needing chronic respiratory care after the episode of ARF support the hypothesis that age-associated lung pathophysiological changes predispose elderly patients to the need for chronic respiratory support when recovering from ARF [14].

Survival of very old patients depended heavily on the context in which NIV was applied. The strong impact of NIV in a context of DNI and *de novo *ARF on 6-month mortality precluded any identification of other risk factors. Development of multivariate analysis in subgroup of patients based on NIV context was not possible because of the limited sample size of our population. In patients with full life support, the use of NIV to reverse *de novo *ARF was associated with a poor outcome. The rate of NIV failure was the highest in this context and hospital mortality was higher than in younger patients. Some studies have suggested a potential increase in mortality associated with NIV failure, in the context of *de novo *ARF [40]. Our results suggest that patients who can benefit from NIV for *de novo *ARF need to be more clearly defined, especially in the very old age group. In addition, in patients with severe chronic respiratory disability, as indicated by previous home respiratory support, long-term mortality was extremely high.

NIV is frequently proposed for very old patients with a DNI decision [23,41]. The outcome of NIV in this context has received little attention and remains controversial [17,42,43]. Hospital mortality was higher in very old DNI patients than in very old patients with full care intensity (56% vs. 27%), but this difference was larger in younger patients (72% in case of DNI vs. 21%). Two previous studies in critically ill DNI patients managed with NIV reported hospital mortality of 57% and 65% [15,18]. Schettino et al. observed that DNI survivors were older than DNI nonsurvivors [18]. This difference suggests different reasons for DNI decisions in elderly and younger patients. Whereas hospital mortality appears acceptable in very old DNI patients, only four patients remained alive at phone interview. One previous study of 34 DNI patients of various ages in whom NIV was applied in the ICU found a 6-month mortality rate of 85% [41]. The survival of DNI patients might depend on the cause of ARF, with a better reported survival at hospital discharge in the case of NIV for CPE and COPD exacerbations [15,18]. In our cohort, 30/39 DNI very old patients received NIV to reverse CPE or COPD exacerbation; 14 were discharged alive from the hospital, but only 2 had survived at 6 months. For DNI patients, physicians can apply NIV with the goal of reversing ARF or for the comfort of patients at the end of life. These two approaches will be associated with different survival rates. An overlap also can exist between these two approaches [43]. A limitation of the study, due to its small sample size, is that we did not separate NIV as a ceiling therapy and NIV indicated for comfort. Very old patients with ARF are frequently unable to discuss and make decisions about their treatment. The DNI decision was based on advance directives in only one patient. Treatment limitations are discussed with family, general practitioners, and are based on decisions of medical and nursing staff. With regard to pre-ICU cognitive, functional, and respiratory status, limitations of endotracheal intubation seemed justified (Table 4). Interestingly, the outcome of very old patients intubated because of NIV failure was no better than that in patients not intubated due to endotracheal intubation limitation. Endotracheal intubation after NIV failure in this population of patients seems of questionable benefit, and further studies should focus on the long-term outcome of this subgroup.

Our study is monocentric and only observational. The frequency and outcome of NIV depend on the expertise of medical and nursing staff in managing this technique. Our study illustrates the NIV practice in a specific ICU, and results cannot be applied for such very old patients managed on the ward.

## Conclusions

This cohort study outlines our experience of NIV as a frequently used ventilatory support in very old patients admitted to the ICU. Importantly, very old patients have similar hospital survival rates compared to younger patients when NIV is applied in validated indications (CPE-AOC respiratory failure and prevention of ARF during the postextubation period), with an acceptable long-term outcome. The use of NIV in a palliative context needs to be further addressed regarding its effects on comfort and outcome, and the outcome after endotracheal intubation in case of NIV failure is particularly poor in this population of patients.

## Abbreviations

ADL: activities of daily living; AOC: acute on chronic; ARF: acute respiratory failure; CPE: cardiogenic pulmonary edema; DNI: do not intubate; ICU: intensive care unit; NIV: noninvasive ventilation; RCT: randomized controlled trial.

## Competing interests

F. Schortgen, A. Follin, L. Piccari, F. Roche-Campo, G. Carteaux, E. Taillandier-Heriche, S. Krypciak, AW. Thille, and E. Paillaud declare that they have no competing interests. L. Brochard, for the past 5 years, has received research grants for clinical trials from the following companies: Maquet (NAVA); Covidien (PAV+); Dräger (SmartCare); General Electric (FRC); Respironics (NIV); Fisher paykel (Optiflow).

## Authors' contributions

FS contributed to the study concept and design, had access to the data, and takes responsibility for the integrity of the data, the accuracy of the data analysis, and the drafting of the manuscript. AF contributed to collecting the data, the design of the study, and data analysis. LP, FRC, and GC contributed to the study concept and design and collecting the data. ETH, SK, EP, and AWT contributed to the study concept and design. LB contributed to the study concept and design and the writing of the manuscript. All authors read and approved the final manuscript.
